# Valorisation of the Invasive Macroalgae *Undaria pinnatifida* (Harvey) Suringar for the Green Synthesis of Gold and Silver Nanoparticles with Antimicrobial and Antioxidant Potential

**DOI:** 10.3390/md21070397

**Published:** 2023-07-09

**Authors:** Noelia González-Ballesteros, Mário Fernandes, Raúl Machado, Paula Sampaio, Andreia C. Gomes, Antonella Cavazza, Franca Bigi, Maria Carmen Rodríguez-Argüelles

**Affiliations:** 1 Departamento de Química Inorgánica, Universidade de Vigo, 36310 Vigo, Spain; noeliagb@uvigo.es; 2 Centre of Molecular and Environmental Biology (CBMA)/Aquatic Research Network (ARNET) Associate Laboratory, Universidade do Minho, Campus de Gualtar, 4710-057 Braga, Portugal; 3 Institute of Science and Innovation for Sustainability (IB-S), Universidade do Minho, Campus de Gualtar, 4710-057 Braga, Portugal; 4 Dipartimento Scienze Chimiche, Della Vita e della Sostenibilità Ambientale, Università di Parma, 43124 Parma, Italy; 5 Institute of Materials for Electronics and Magnetism, National Research Council, 43124 Parma, Italy

**Keywords:** *Undaria pinnatifida*, green synthesis, silver nanoparticles, gold nanoparticles, antibacterial activity, antifungal activity

## Abstract

Bacterial and fungal infections are a challenging global problem due to the reported increasing resistance of pathogenic microorganisms to conventional antimicrobials. Nanomaterials are a promising strategy to fight infections caused by multidrug-resistant microbes. In this work, gold (Au@UP) and silver (Ag@UP) nanoparticles were produced for the first time by green synthesis using an aqueous extract of the invasive macroalgae *Undaria pinnatifida* (UP). The nanoparticles were characterized by a wide range of physicochemical techniques. Au@UP and Ag@UP demonstrated to be spherical and crystalline with an average size of 6.8 ± 1.0 nm and 14.1 ± 2.8 nm, respectively. Carbohydrates and proteins of the UP extract may participate in the synthesis and capping of the nanoparticles. The UP extract, Ag@UP, and Au@UP were assessed for their antimicrobial activity against *Escherichia coli*, *Staphylococcus aureus, Pseudomonas aeruginosa, Candida albicans*, and *Candida auris.* Ag@UP showed the highest antimicrobial activity with very low MIC and MBC values for all the tested bacteria, and Au@UP demonstrated to be very effective against biofilm-producing bacteria. The antifungal properties of both Ag@UP and Au@UP were remarkable, inhibiting hyphae formation. This study points towards a very promising biomedical exploitation of this invasive brown algae.

## 1. Introduction

Antimicrobial resistance is one of the ten global public threats facing humanity and is considered by the World Health Organization as a priority issue [1,2]. Bacterial and fungal infections are a common worldwide problem, which is becoming increasingly challenging due to the reported increasing resistance to treatment in several microbial pathogens causing common infections in the community. Biofilm-forming bacteria are recognized as a bigger threat, as biofilm-residing cells can be impervious to the host’s immune system, antibiotics, and other treatments, playing a role in certain pathogenesis [3].

Among the nineteen pathogens identified as priority groups by the World Health Organization (WHO) in its first Fungal Priority Pathogens List—WHO FPPL, published in 2022—*C. albicans* and *C. auris* are identified in the critical priority group [4,5]. *Candida* species are opportunistic pathogens that cause infections in immunocompromised hosts [6]. Invasive candidiasis rank among the most prevalent nosocomial blood infections [7], with the most severe forms of infection reaching a mortality above 70% [6]. *C. albicans* can rapidly acquire resistance to the most frequently used antifungals, such as fluconazole. On the other hand, *C. auris* is an emerging fungus considered an urgent antimicrobial resistance threat and is responsible for serious hospital outbreaks. The exponential development in recent years of multidrug resistance, combined with the scarcity of antifungal drugs due to the difficulty in ensuring selectivity in relation to animal cells, has propelled research on metal nanoparticles as potential alternatives [8]. Similarly, the bacteria *Escherichia coli*, *Pseudomonas aeruginosa*, and *Staphylococcus aureus*, belonging to the ESKAPE group of pathogens, are among the most challenging antibiotic-resistant pathogens [9]. These bacteria are common opportunistic pathogens in nosocomial infections and are associated with a high risk of mortality and high economic burden [9,10].

Nanomaterials have arisen as a promising solution in the fight against infections thanks to their outstanding physicochemical properties [11,12]. Nanoparticles present a high surface-to-volume ratio, which potentiates reactivity and interaction with microorganisms and may also present different sizes and shapes, which has a direct impact on their bioactivity [13,14]. A wide variety of nanoparticles have an inherent bactericidal activity through different possible mechanisms [15]. This is the case of silver nanoparticles (AgNPs), which have been extensively investigated due to their broad-spectrum antimicrobial activity [16,17], and gold nanoparticles (AuNPs), which, although less investigated, in recent years, have been increasingly associated with antimicrobial activity [18,19]. The possibility of combining into one nanomaterial strong antibacterial and antifungal activities would be thus invaluable to control infections.

Continuing our research on the use of macroalgae as bionanofactories, *Undaria pinnatifida* (Harvey) Suringar [20] has been selected as the biogenic precursor for the green synthesis of metal nanoparticles. *U. pinnatifida* is a brown kelp-like algae native to northeast Asia, and its macromolecular content makes it interesting for nanoparticle synthesis. It is a winter annual species that inhabits rocky substrates from the low intertidal to an 18 m depth. It has a global non-native range and is considered one of the world’s “worst” invasive species [21,22], featured on the list of the top 10 invasive species in Europe [23]. *U. pinnatifida* is considered the most widely eaten brown seaweed due to its high amounts of proteins, polysaccharides, and fucoxanthin. Thus, its biotechnological exploitation may have a positive impact in terms of bio-sustainability and biodiversity. Furthermore, UP contains valuable pharmacological compounds with several biological activities, such as the prevention of hyperglycaemia, the suppression of chemically induced mammary tumours, or the inhibition of antihypertension [24]. Recently, it has also been revealed that polysaccharides extracted from *U. pinnatifida*, such as fucoidan, exhibit antibacterial activity [25].

In this study, gold and silver nanoparticles, produced by green synthesis with the *Undaria pinnatifida* (UP) extract, were tested for their antimicrobial activity against bacteria of clinical relevance (*E. coli*, *P. aeruginosa*, and *S. aureus*), including their capacity to form biofilms, and on two *Candida* species (*C. albicans* and *C. auris*) to infer their antibacterial and antifungal potentials.

## 2. Results and Discussion

### 2.1. Synthesis and Characterization of Gold and Silver Nanoparticles

In the present study, the UP aqueous extract was employed for the reduction of Au(III) and Ag(I) into AuNPs and AgNPs. An optimization process for the synthesis was performed to attain nanoparticles homogeneous in size and shape. With this aim, numerous reaction conditions were assessed by modifying the concentration of the UP extract, the concentration of the metal salt employed (either HAuCl_4_ or AgNO_3_), and the temperature of reaction. In all cases, the reactions were monitored by colour changes and UV-Vis spectroscopy.

**Figure 1:**
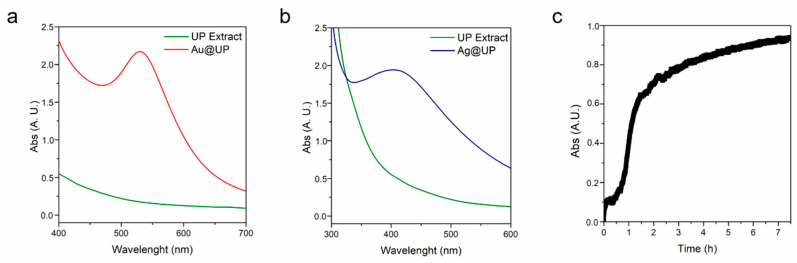
UV-Vis spectra of UP extract before and after the synthesis of (**a**) Au@UP and (**b**) Ag@UP. (**c**) Time-course spectra of the synthesis of Au@UP.

Regarding the synthesis of Au@UP, a colour change from pale brown to red was observed when gold nanoparticles were produced. The best reaction conditions were found to be a concentration of the extract of 1 g/mL, 0.4 mM of HAuCl_4_, and conducted at room temperature for 24 h. Figure 1a depicts the UV-Vis spectrum of the UP extract. The lack of absorption in the wavelength range represented can be noted; however, after the production of Au@UP, a band with maximum of absorbance (λ_max_) at 530 nm appears, corresponding to the characteristic surface resonance (SPR) band of AuNPs.

Concerning Ag@UP synthesis, a colour change from pale brown to yellow/orange was noted; however, when performing the synthesis at room temperature, the reaction took long periods of time (up to 48 h), and the nanoparticles were heterogeneous in size. When increasing the temperature to 100 °C, the reaction was faster, resulting in the characteristic SPR band of AgNPs at 411 nm after only 8 h (Figure 1b). The final reaction conditions for the synthesis of Ag@UP were 0.5 g/mL of the extract, 0.17 mM of AgNO_3_, and conducted at 100 °C for 8 h. These reaction conditions were employed for the preparation of the samples hereafter.

Regarding the reaction time in Au@UP synthesis, absorbance measurements were acquired at λ_max_ 530 nm after the addition of HAuCl_4_ to the UP extract. The spectrum obtained can be divided in three main stages (Figure 1c). The first is an activation step, corresponding to the first hour after the addition of the gold salt, wherein no visual changes were observed. Then, an increase in absorbance appears between 1 and 2 h, corresponding to the observed change in colour and the time when the nucleation process might take place. Finally, the reaction slowed down, and the measurements were stopped after 8 h.

Z potential measurements were acquired to study the charge and stability of Au@UP and Ag@UP, obtaining values of −16.9 ± 2.2 and −27.7 ± 1.2 mV, respectively. These results indicate that both samples form a stable colloidal suspension with the particles carrying a negative electrostatic surface charge.

**Figure 2:**
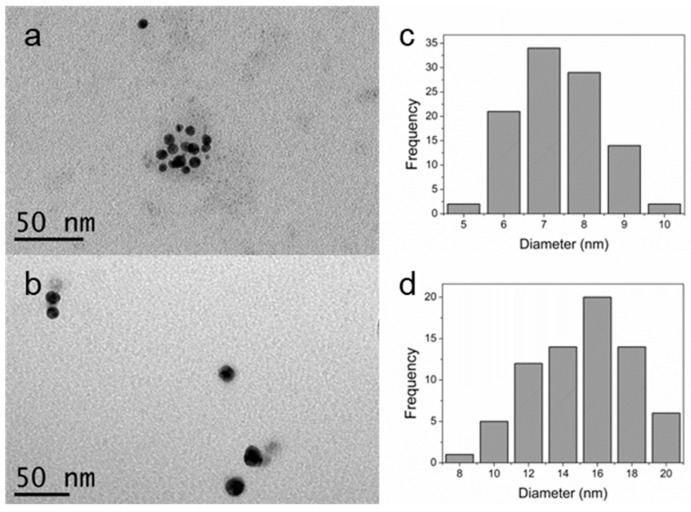
(**a**,**b**) Transmission electron microscopy image and (**c**,**d**) size distribution histogram of Au@UP and Ag@UP, respectively.

Figure 2a,b collects the transmission electron microscopy images acquired for Au@UP and Ag@UP, respectively. It can be observed that, in both cases, NPs are spherical. Figure 2c,d shows the size distribution histogram after the measurement of >100 nanoparticles from TEM images, and the results indicate a mean size of 6.8 ± 1.0 nm for Au@UP and 14.1 ± 2.8 nm for Ag@UP. It is noteworthy to highlight the small size observed for the Au@UP nanoparticles. Stable nanoparticles with diameters inferior to 10 nm are quite difficult to obtain, and normally, a second step for the stabilisation of the NPs is required since they tend to aggregate and form clusters. In the present study, a one-step procedure for the synthesis is proposed.

**Figure 3:**
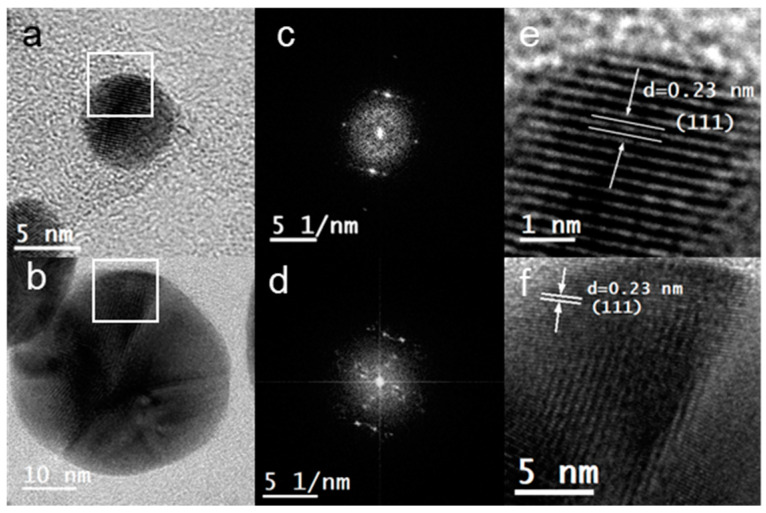
(**a**,**b)** High resolution image, (**c**,**d**)Fourier transform, and (**e**,**f**) amplification of the marked area of (**a**,**b**) of Au@UP and Ag@UP, respectively.

High Resolution TEM (HRTEM) was also performed to study the crystallinity of the samples. Figure 3a,b shows HRTEM images of Au@UP and Ag@UP, respectively. It can be observed in the images obtained that the particles display an internal complex contrast, and the Fourier transform (Figure 3c,d) confirmed their crystallinity. The d-spacing was calculated in the marked area of Figure 3a,b. Then, tabulated data allowed to assign the corresponding Miller index. In the case of Au@UP (Figure 3e), a d-spacing of 0.23 nm was measured, which corresponds to Miller index (111). Regarding Ag@UP (Figure 3f), the preferential d-spacing calculated for the NPs was also 0.23, which corresponds to Miller index (111). In both cases, the results are consistent with a face-centred cubic crystalline structure for gold and silver.

**Figure 4:**
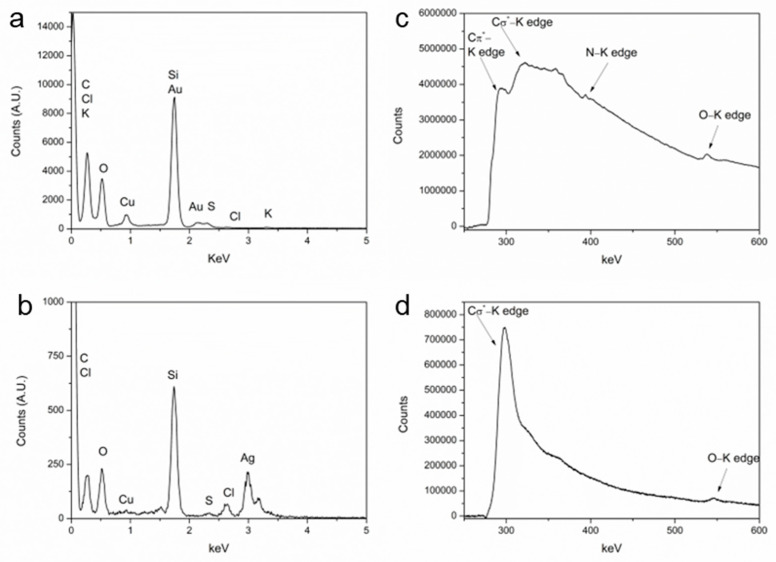
(**a**,**b**) Energy dispersive X-ray analysis of Au@UP and Ag@UP, respectively. Electron energy loss spectroscopy spectrum of (**c**) Au@UP and (**d**) Ag@UP.

**Figure 5:**
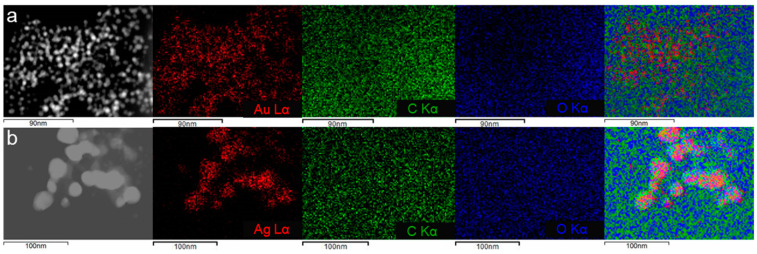
Dark field scanning transmission electron microscopy image, gold or silver, carbon and oxygen mapping, and mix map of (**a**) Au@UP and (**b**) Ag@UP.

An energy dispersive X-ray analysis was performed (Figure 4a,b) in the area shown in the scanning transmission electron microscopy images that appears in Figure 5a,b. Apart from gold and silver in the corresponding samples, the presence of other elements in the seaweed was confirmed. In both samples, the elements present were carbon, chlorine, sulphur, and oxygen. The spectrum of Au@UP also showed the presence of potassium. These results are in accordance with other studies related to the composition of *U. pinnatifida* [26]. The copper signal that appears in all spectra can be due to the copper grids and to the composition of the seaweed. In fact, several studies have stated that copper is found in brown seaweed as a trace element [26,27,28].

Electron energy loss spectroscopy (EELS) (Figure 3c,d) spectra were acquired, since this technique is better than EDX for the detection of light elements (atomic number <11). Furthermore, to eliminate the contribution of the grids in the detection of the elements, the analysis was performed in areas over a hole in Holey carbon support films. In the EELS spectra of Au@UP (Figure 3c), the characteristic edge of the carbon K edge (284 KeV), the nitrogen K edge (401 KeV), and the oxygen K edge (532 KeV) were observed. In the case of the C edge, this technique even allows to differentiate between π* and σ* bonds. However, regarding Ag@UP’s EELS spectra (Figure 3d), only the edges of carbon and oxygen appeared, probably as a consequence of the lower concentration of extract employed during the synthesis as compared with Au@UP.

In Figure 5a,b, the elemental mapping of the samples built from the EDX spectra in the area identified in the STEM image are shown, selecting carbon (green), oxygen (blue), and the metal (red). In both cases, the results confirmed that carbon and oxygen surround the metal nanoparticles, while gold and silver are concentrated in the particles, which suggests the complete reduction of the salts employed.

All the assays performed allow to propose that the nanoparticles are surrounded by the UP extract which acts as both a reducing and protective agent, preventing their aggregation and precipitation.

**Figure 6:**
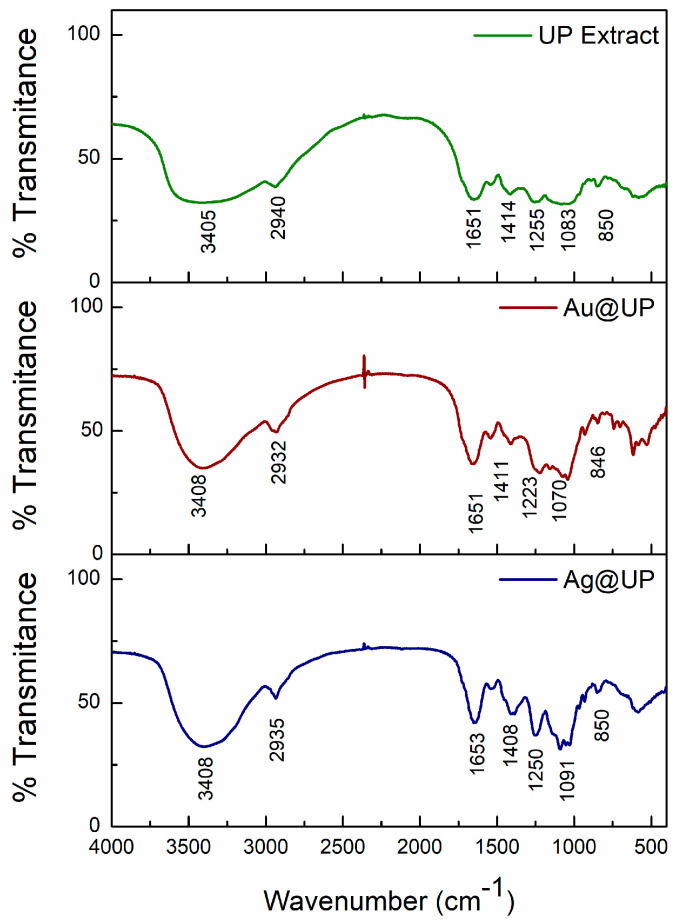
Fourier transform infrared spectra of UP extract before and after the synthesis of Au@UP and Ag@UP.

Fourier transform infrared spectroscopy (FTIR) was acquired to understand which functional groups and molecules of the UP extract are involved in the reduction and stabilization of Au@UP and Ag@UP. Firstly, it is important to highlight that the UP extract is a complex mixture of the hydrosoluble components of *U. pinnatifida.* It is expected that the FTIR spectra possess a contribution of the main components, such as polysaccharides and proteins, with slight alterations due to the presence of other minority components. The spectra obtained are collected in Figure 6, and the assignment of the bands was made based on previous reports both for the *U. pinnatifida* extracts and the extracted polysaccharides [29,30,31].

The first region of the spectra between 4000 and 2000 cm^−1^ shows two bands present in all seaweed extracts. The first band at 3405 cm^−1^ in the spectrum of the UP extract is assigned to a hydroxy stretch in intermolecular-bonded alcohols or even residual moisture. These moieties can be found in carbohydrates and polyphenols, among other compounds. The second weak band centred at 2940 cm^−1^ is typically assigned to aliphatic CH stretching vibrations, seen in many compounds [32,33].

In the second region between 1700–1400 cm^−1^ appears two main bands, mostly associated with the presence of proteins/amino acids in the extract. First, the intense band at 1651 cm^−1^ can be attributed to carboxylic acid C=O (stretching) and amine N-H (bending), while the weaker band at 1414 cm^−1^ can be assigned to methyl and methylene C-H (bending), carboxylic acid O-H (bending), and alcohol O-H (bending) [34].

Finally, the sequence of bands that appears in the region between 1200–800 cm^−1^ is characteristic to the presence of polysaccharides in seaweed extracts. Particularly, the wide band at ~1083.0 cm^−1^ could be assigned to the C-O and C-C stretching vibrations of the pyranose ring [33]. The presence of the intense band at 1255 cm^−1^ and a weaker one at 842 cm^−1^ confirms the presence of sulphated polysaccharides, such as fucoidan, in the extract. These bands are associated with the asymmetric O=S=O stretching vibration of sulphate esters to the COS bending vibration of sulphate substituents at axial C4 positions, respectively [30].

When comparing the spectra of the UP extract before and after the synthesis of Au@UP and Ag@UP (Figure 6), minor shifts can be noted in the position and intensity of the bands at 3400 and 1600 cm^−1^; meanwhile, the most relevant differences are observed in the region between 1200–800 cm^−1^. In particular, the shift of the band assigned to the sulphate group of polysaccharides from 1255 to 1223 cm^−1^ in Au@UP can be highlighted. In summary, these results suggest the participation of polysaccharides and proteins; however, the participation of other molecules in the reducing and stabilizing process of the synthesis of the NPs cannot be excluded.

### 2.2. Carbohydrate Analysis

Carbohydrates are one of the main class of compounds occurring in algae and can represent structural polysaccharides constituting the cell wall composition or can be present in plastids. The amount and chemical nature of their dietary fibre content affect the functional properties of seaweeds in terms of the technological properties of their extracts, influencing texture and viscosity, and also their potential health effects. In *U. pinnatifida,* polysaccharides have been reported to reach up to 35% of the dry weight, specifically, 30 g/100 g of soluble fibres and 5.3 g/100 g of insoluble fibres [35].

Previous studies report that raw materials derived from different plant tissues, as well as harvesting time, have a significant impact on the final extraction yield [25]. The main sugars detected after hydrolysis were fucose, rhamnose, glucose, galactose, xylose, and mannose. In addition, glucuronic acid, galacturonic acid, arabinose, uronic acid, and sulphate were detected. The average molecular weights of *U. pinnatifida* carbohydrates were determined by several techniques, including HPLC, and were found to range from ∼1.0 × 10^3^ Da to ∼7.5 × 10^5^ Da–3.7 × 10^4^ Da and 1.1 × 10^5^ Da [25]. However, data from the literature did not provide punctual information about the composition and the occurring chain length of the molecules belonging to the multidispersed mixture.

**Figure 7:**
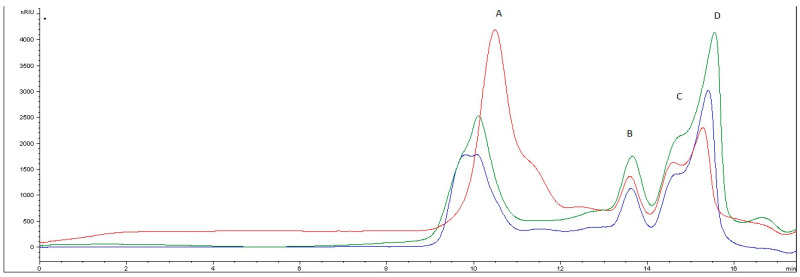
Chromatographic profiles of *U. pinnatifida* extract before (blue line) and after Ag@UP (red line) and Au@UP (green line) formation.

The analyses performed in this study, using size exclusion chromatography, allowed the fractionation of the carbohydrate mixtures and separated different bands. The chromatographic profile obtained is shown in Figure 7, where the presence of multiple bands indicates the groups of analytes characterised by different molecular weights. The retention time of the dextrans employed as standards permitted to build a calibration curve, allowing the calculation of the different mass weight values: a first band (peak A), eluting at about 10 min, corresponds to a MW higher than 150 kDa; peak B, eluting at about 13 min, corresponds to approximately 72 kDa; peaks C and D, eluting at about 15 and 16 min, correspond to 32 kDa and 14 kDa, respectively.

Some differences can be noticed between the three profiles reported in the Figure, related to the original extract (blue line) and those obtained after Ag and Au formation (red and green line, respectively).

Specifically, after formation of Ag@UP, small qualitative differences could be detected, mostly related to the shift of retention time of the first fraction moving towards lower MW values. From the quantitative point of view, a semiquantitative approach based on the estimation of the area values of the different peaks confirmed an increase of the first peak (A), suggesting a possible chain-length shortage. In parallel, a reduction of the last bands (B, C, and D) was recorded.

As for the formation of Au@UP, the profile did not show sensible variations, but a slight difference in the shape of the first fraction (A) can be evidenced, accompanied by an increase of the last band. This variation can also suggest the formation of species with MWs lower than the initial ones.

These data suggest an involvement of the carbohydrates in the formation process of the nanoparticles, confirming the importance of this class in stabilising them, as already reported in previous studies [36,37].

### 2.3. Antioxidant Activity

**Figure 8:**
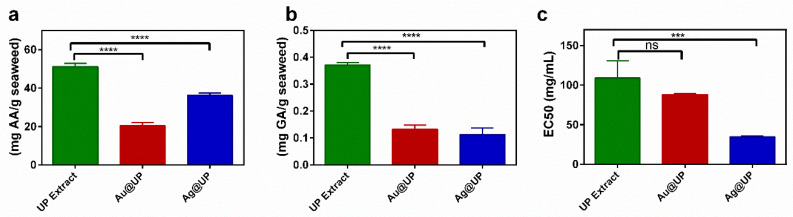
Graph bars of (**a**) the reducing power, (**b**) total phenolic content, and (**c**) DPPH scavenging activity of the UP extract, Au@UP, and Ag@UP. In the graphs: ns *p* > 0.05, *** *p* ≤ 0.001, **** *p* ≤ 0.0001.

The antioxidant potential of the *U. pinnatifida* aqueous extract was determined by three assays, the reducing power, the total phenolic content, and the DPPH scavenging activity (Figure 8).

Firstly, the UP extract presents a reducing power value of 51.2 ± 1.6 mg AAE/g seaweed. There are some previous reports on the reducing power of this species; however, results are expressed as optical density [38] or as IC_0.5_ [39], so a comparison is not possible. When comparing this result with the ones obtain for other brown seaweed aqueous extracts obtained and analysed by the same methods, it was observed that UP presents a lower reducing capacity than *C. baccata* [40], *S. polyschides* [41], *S. muticum* [42], *D. antarctica* [43], and *D. menziesii* [44]; nevertheless, this reducing capacity is sufficient to synthesize gold and silver nanoparticles. Other works also confirmed the observation that UP possesses the lowest reducing power among other brown seaweeds [45,46].

Regarding TPC, the UP extract possesses a value of 0.37 ± 0.01 mg GAE/g seaweed. The data obtained in the literature for *U. pinnatifida* is miscellaneous, with some studies obtaining high values of TPC, while the values obtained in others are much lower or cannot be compared [38,39,47]. These differences show how the extraction technique, the solvents, and times employed as well as the various determination methods affect the results obtained.

These dissimilarities were also observed in the case of the DPPH assay, as a value of 109.9 ± 21.8 mg/mL was obtained, while in the literature, some studies provide the results as a percentage of inhibition [45,47]. There are also some studies that do not indicate the concentration tested [38]. An interesting study analysed how the different sulphate content in polysaccharides extracted from *U. pinnatifida* affects the percentage of inhibition of DPPH, observing that a higher sulphate group content of polysaccharides indicates a stronger DPPH-free radical scavenging capacity at the same concentration [46].

These assays were also performed after the synthesis of the nanoparticles. Regarding reducing power, a significant decrease in the values obtained can be noted. Au@UP and Ag@UP possess, respectively, 2.5 and 1.5 times less reducing power than the UP extract. Regarding TPC, a significant decrease of three times in Au@UP and Ag@UP was observed. This suggests an active role of the phenolic compounds present in the UP extract in the reduction process for the synthesis of the NPs. In the case of the DPPH, no significant difference was observed in Au@UP, while an increase in the scavenging potential of Ag@UP was noted. A similar behaviour has been previously observed in other gold and silver nanoparticles synthesized by brown seaweeds, where higher DPPH scavenging activity was obtained in silver nanoparticles, and no significant differences or lower activity in gold nanoparticles [41,42] was observed.

### 2.4. Antibacterial Activity of UP Extract, Ag@UP and Au@UP

The antibacterial activity of Ag@UP and Au@UP, as well as of the UP extract, was evaluated by determining the minimum inhibitory concentration (MIC) and minimum bactericidal concentration (MBC) against the pathogenic bacteria *Escherichia coli* ATCC 11303 (gram-negative), *Pseudomonas aeruginosa* ATCC 10145 (gram-negative), and *Staphylococcus aureus* ATCC 6538 (gram-positive).

The Ag@UP nanoparticles exhibited potent antibacterial activity against all the tested bacteria, with MIC and MBC values of 1.96 μg/mL and 3.94 μg/mL, respectively (Table 1). On the other hand, no measurable MIC or MBC values were observed for the Au@UP nanoparticles, even at the highest tested concentration of 11.81 μg/mL. Notably, the Ag@UP nanoparticles demonstrated to be even more potent than the antibiotics used as a control (ampicillin and kanamycin; MIC of 40 μg/mL for *E. coli* and 60 μg/mL for *P. aeruginosa* and *S. aureus*), reaching MIC values more than 20 times lower and an average of 5 times lower, compared with silver nitrate (Table 1) [48]. Few reports associate UP extracts with antibacterial activity, which is mostly due to interference of fucoidans with bacterial adhesion to human cells in culture [25]. Therefore, it is not surprising that the MIC values of the UP extract against the tested bacteria were not measurable, at least up to the concentration of 1700 μg/mL.

The ability of the biosynthesized nanoparticles to inhibit biofilm formation was evaluated using *P. aeruginosa* PAO1 and *S. aureus* ATCC 25923 as model microorganisms [48]. While the UP extract had no inhibitory effects, both Ag@UP and Au@UP displayed an inhibitory effect at concentrations lower to the reference antibiotics (Table 2). Comparing with the values obtained for planktonic cells (Table 1), a higher concentration of Ag@UP nanoparticles was required to inhibit biofilm formation, with effective inhibition observed only at a concentration of 7.88 μg/mL (Table 2). Interestingly, although no MIC was observed for the Au@UP nanoparticles against planktonic cells, these nanoparticles demonstrated an inhibitory effect against the biofilm-forming bacteria at a concentration of 11.81 µg/mL (Table 2).

Although there are several reports on the antibacterial activity of gold and silver nanoparticles produced by green synthesis in brown macroalgae, very few compare their effects on planktonic versus biofilm-producing bacteria. Moreover, the methodologies are not standardized regarding the tested microorganisms and often resort to the quantification of inhibition halos, resulting in difficult a quantitative comparison. Nevertheless, some comparisons can be drawn which support the advantages of the nanoparticles included in this study. Comparing the results of the present study with silver and gold nanoparticles produced with extracts of the brown seaweeds *Cystoseira tamariscifolia* and *Cystoseira baccata*, the MIC values for the non-biofilm-producing bacteria are at much lower concentrations with the biogenic *U. pinnatifida* nanoparticles [48]. Other authors reported that silver nanoparticles synthesized in *Sargassum wightii* (average size 48.78 nm) were toxic to *S. aureus* at a MIC of 130 µg/mL [49], while particles produced in *Turbinaria conoides* (up to 80 nm) showed a MIC of 64 and 8 µg/mL against *P. aeruginosa* and *S. aureus*, respectively [50]. Gold nanoparticles produced in *Sargassum illicifolium* (20–25 nm) and in *Sargassum muticum* (10.4 ± 1.2 nm) registered a MIC in planktonic *S. aureus* of 48 and 39.5 µg/mL, respectively [42,51]. Interestingly, Salam et al. (2020) reported a MIC of 25 µg/mL for biofilm-producing *S. aureus*, with 10–20 nm gold nanoparticles produced in *Padina tetrastromatica* [52]. Altogether, the results presented here are very promising, considering the low concentrations necessary to kill the tested bacteria, particularly with Ag@UP, even in biofilm-producing strains.

### 2.5. Antifungal Activity Assessment of UP Extract, Ag@UP and Au@UP

Antifungal assays were performed against two *Candida* species, *C. albicans* and *C. auris*. The activity of these NPs was tested in clinical isolates to evaluate their potential antifungal activity (Table 3). Again, the UP extracts did not present activity in the tested concentrations, and to our knowledge, there are no published studies on the antifungal effects of UP. The minimum fungicidal concentration (MFC) determined for both Ag@UP and Au@UP are at very low concentrations, interestingly more striking for the strains with less sensitivity to fluconazole, which is the most widely used antifungal agent previously determined [53] to have a MFC of 0.125 μg/mL on *C. albicans SC5314,* >64 μg/mL for *C. albicans 124a*, and above 128 μg/mL for both *C. auris* isolates. The *C. auris* isolates were also determined to be more sensitive to micafungin [54] (MFC 0.25 μg/mL) in comparison to *C. albicans* SC5314 (MFC 0.016 μg/mL).

To understand if the nanoparticles affected the ability of the yeast cells to form hyphae, a major virulence determinant, hyphae formation assays were performed with the four *Candida* strains (Supplementary Materials). This parameter is not often studied, despite offering a complementary perspective on the possible mechanism of the actions of antifungal nanoparticles. The underlying mechanisms by which some natural compounds, such as piperine in *P. nigrum*, interfere with *Candida*’s transition to hyphae-forming cells are still not well understood [55], but they should affect this pathogen’s capacity to adhere and invade host tissues. The extract itself did not affect this morphological transition, but all nanoparticles led to a notorious impairment in hyphae growth, which indicates their positive action towards limiting infections and in the strains least susceptible to fluconazole.

The antifungal activity of plant-synthesized AgNPs is widely recognized, but few studies report this activity of NPs synthesized in extracts of macroalgae. Focusing on brown alga, toxicity against *C. albicans* was only reported with gold nanoparticles produced in *Turbinaria ornata* and *Padina tetrastromatica* [56] and with silver nanoparticles from *Lobophora variegata* [57], *Saccharina japónica* [58], *Sargassum longifolium* [59], *Sargassum polycystum* [60], and *Turbinaria ornata* [56], with varying sizes of inhibition halos achieved. Only the study by Thiurunavukkaran et al. (2022) calculated the MIC, although only a 15% kill was reported for *C. albicans* at 32 μg/mL [60], which is very modest compared to the results presented here. A much stronger effect was reported with gold nanoparticles produced in microalgae *Spirulina*, with a MIC of 32 μg/mL [61]. In addition, other authors in the past seldom evaluated antifungal activity using isolates of known resistance to conventional treatments, even though finding treatments for multidrug resistance microbes is now a major concern.

Similar sized AgNPs biosynthesized in plants or fungi have been reported to possess an effect on *C. albicans*. In most reports, reference strains are used and often differ between studies, which also renders rigorous comparisons difficult. Approximate or lower MICs were obtained only with few *Candida albicans* strains for AgNPs produced in *Arthroderma fulvum* (*C. albicans* ATCC90028, MIC 0.5 μg/mL), *Aspergillus sydowii* (*C. albicans* ATCC90028, MIC 0.25 μg/mL), and *Epicoccum nigrum* (*C. albicans* ATCC90028, MIC 0.5 μg/mL) [8].

To the best of our knowledge, no other reports are available regarding *C. auris* exposed to green-synthesized gold or silver nanoparticles.

## 3. Materials and Methods

### 3.1. Seaweed Collection and Extract Preparation

*Undaria pinnatifida* (Harvey) Suringar (UP) samples were collected in the north-west coast of Spain (42°12′2.9″ N; 8°47′6.2″ W) at the lower intertidal rocky shore. Samples were collected and taxonomies were identified by Professor Mariano Lastra-Valdor from the UVIGO Marine Research Centre (CIM-UVIGO) (Figure S3, supplementary material). UP samples were brought to the lab and were either immediately processed or frozen at −24 °C until treatment.

For the extract preparation, the protocol previously reported with other seaweeds was followed [40,62]. Briefly, when needed, the macroalgae were defrosted and rinsed with water to remove possible contaminations, and excess water was removed, extending the fronds on blotting paper. Then, the sample was cut into fine pieces, and a hot water extraction was performed employing Milli-Q water in a proportion of 1 g of seaweed per mL of water. The mixture was then boiled at 100 °C for 15 min. Once the extraction was completed, the extract was separated from the remaining biomass by filtration and was centrifuged at 4500 rpm for 20 min, and the supernatant was filtered again.

### 3.2. Synthesis of Gold and Silver Nanoparticles

For the optimization of the synthesis of gold and silver nanoparticles led by the UP extract, the protocol reported for other seaweeds was employed [37,41]. Different concentrations of the extract and the metal salts at different temperatures were tested. The reactions were monitored by colour changes and UV-Vis spectroscopy until the obtainment of homogeneous nanoparticles with narrow size distributions, as confirmed by TEM. The best conditions for obtaining the nanoparticles are briefly described below.

In the case of Au@UP, an extract concentration of 1 g/mL was kept at room temperature (RT) under stirring, and HAuCl_4_ was added slowly, obtaining a final gold concentration of 0.4 mM. The reaction was kept under stirring for 24 h.

Regarding Ag@UP, an extract concentration of 0.5 g/mL and a final silver concentration of 0.25 mM were used. The extract was heated at 100 °C, and then AgNO_3_ was added while stirring. The mixture was kept under reflux and stirring for 30 min.

### 3.3. Characterization Techniques

A UV-Vis spectra characterization of the UP extract, Au@UP, and Ag@UP was performed in a Jasco Spectrometer V-670 670 (JASCO, Tokyo, Japan) between 200–700 nm at room temperature.

The concentration of gold and silver was determined by Inductively Coupled Plasma Optical Emission spectroscopy (ICP-OES) using a Perkin Elmer Optima-4300 DV (Perkin Elmer, Inc., Waltham, MA, EEUU) with Indium as the internal standard.

Zeta potential measurements of the Au@UP and Ag@UP colloidal suspension were acquired in a ZetasizerNano S (Malvern Instruments, Malvern, UK) equipped with a He–Ne laser (wavelength 633 nm) and backscatter detection (173°) by taking the average of five measurements at the stationary level.

Electron microscopy samples were prepared following a purification step to eliminate part of the organic fraction. Thus, the nanoparticle solutions were centrifuged at 10,000 rpm for 30 min, and the pellets were dispersed in Milli-Q water and sonicated for 15 min. A drop of the nanoparticles’ dispersions was place onto copper grids.

A JEOL JEM 1010 (100 kV) microscope (JEOL, Tokyo, Japan) was employed for the acquisition of the TEM images, while a JEOL JEM2010F field emission gun TEM (JEOL, Tokyo, Japan), operated at 200 kV, was employed for the HRTEM and STEM images. Coupling between the STEM unit and the EDS detector (Oxford Inca Energy 200 Oxford Instruments Analytical, High Wycombe, UK) was used to obtain elemental maps. EELS spectra were recorded in STEM mode using a Gatan Quantum EELS GIF with a collection semi-angle of β = 16.75 mrad. The energy resolution was ~1.75 eV (FWHM of the zero-loss peak). In all cases, data collection and analysis were carried out using Digital Micrograph software by Gatan, and in the case of EDS, INCA Energy software was also used.

FTIR samples were prepared by drying the UP extract, Au@UP, and Ag@UP in an oven at 80 °C. Then, the samples were ground to obtain a fine powder and employed to record the spectra using the KBr pellet technique. A Jasco FT/IR-6100 spectrophotometer (JASCO, Tokyo, Japan) was employed for the acquisition of the FTIR spectra of the samples in the range of 4000–400 cm^−1^, performing 60 scans at a resolution of 4 cm^−1^ and a scan speed of 2 mm/sec.

### 3.4. Carbohydrate Analysis

The polysaccharide fraction of the extracts was analysed by performing a separation of the different molecules based on size exclusion liquid chromatography. An Agilent 1200 Series instrument equipped with a PL aquagel-OH 40 column (300 × 7.5 mm) with a particle size of 8 µm coupled to a refractive index detector (Agilent 1260 Infinity, Palo Alto, CA, USA) was used (Agilent Technologies, Palo Alto, CA, USA). The experimental conditions for elution are reported in a previous article [37].

Solutions submitted to analysis were obtained by dissolving the dried extracts in water at a concentration of 600 ppm. All solutions were then centrifuged and purified to remove possible interfering substances, such as polyphenols, by elution through a Dionex OnGuard II P cartridge. Prior to analysis, samples were filtered with nylon filters (0.2 micron). All analyses were performed in triplicate, by injecting a volume of 100 microliters.

Solutions of standard dextrans and of a molecular weight of 150, 50, and 12 kDa were used as references for building a calibration curve aimed at the determination of the molecular weight of the separated carbohydrates’ fractions.

### 3.5. In Vitro Antioxidant Activity

The antioxidant activity of the UP extract before and after the synthesis of Au@UP and Ag@UP was performed by three different colorimetric assays, reducing power, total phenolic content, and DPPH radical scavenging activity, following the protocols previously reported [40,44].

#### 3.5.1. Reducing Power

A total of 1.0 mL of the sample (or water for the blank control), 2.5 mL of the phosphate buffer (0.2 M, pH 6.6), and 2.5 mL of potassium ferricyanide (1%) were mixed and incubated at 50 °C for 20 min. Then, the reaction was stopped, adding 2.5 mL of trichloroacetic acid (10%), and the solution was centrifuged at 4000 rpm for 10 min. Finally, 2.5 mL of the supernatant was mixed with 2.5 mL of Milli-Q water and 0.5 mL of ferric chloride (0.1%), turning the solution from yellow to blue. The absorbance was then measured at 700 nm. A calibration curve was built, employing ascorbic acid as the reference (50–400 mg/L). Results are the mean of three independent assays and are expressed as ascorbic acid equivalents (AAE) per gram of seaweed.

#### 3.5.2. Total Phenolic Content

For the quantification of TPC, 100 µL of the sample (or water for the blank control) were mixed and left to stand for 2 min at room temperature with 2 mL of sodium carbonate 5%. Then, 100 µL of the Folin–Ciocalteu reagent 50% was added, mixed thoroughly, and allowed to stand at room temperature in the dark. After 30 min, the absorbance was measured at 725 nm. Gallic acid was selected to build a calibration curve (0.05–1 mg/mL). All measurements were performed in triplicate and are expressed as gallic acid equivalents (GAE) per gram of seaweed.

#### 3.5.3. DPPH Scavenging Activity

A total of 1 mL of DPPH (0.1 mM in methanol, freshly prepared) was added and thoroughly mixed into 3 mL of the extract (previously diluted at a ratio of 1:12). For this assay, it was necessary to prepare a blank, using 3 mL of Milli-Q water instead of the sample, and a sample control by mixing 3 mL of the sample with 1 mL of methanol instead of the DPPH solution. All the mixtures were allowed to stand at room temperature for 30 min. Then, the absorbance was measured at 517 nm. The DPPH radical scavenging activity was calculated with the following formula:

$$DPPH\;scavenging\;effect\;\left(\%\;inhibition\right)=\left(1-\frac{A_{s}-A_{s0}}{A_{b}}\right)*100$$

where *A_s_* is the absorbance of the samples, *A_s_*_0_ is the absorbance of the sample control, and *A_b_* is the absorbance of the blank. All the tests were performed in triplicate. The results are expressed as the concentration required to inhibit the radical concentration of *DPPH* by half (IC_50_).

Ascorbic acid was used as the positive control, obtaining an IC_50_ of 2.5 mg/L.

#### 3.5.4. Statistical Analysis

GraphPad Prism 6 software was employed for the determination of significant differences between the antioxidant activity obtained for the extract before and after the synthesis of Au@UP and Ag@UP, by performing a one-way analysis of variance (either an ANOVA or a Kruskal–Wallis test) and a Tukey’s or Dunn’s test afterwards. All experiments were performed three times. In the graphs, results are expressed as ns *p* > 0.05, * *p* ≤ 0.05, ** *p* ≤ 0.01, *** *p* ≤ 0.001, and **** *p* ≤ 0.0001.

### 3.6. Antibacterial Activity

The antibacterial performance of the *Undaria pinnatifida* (UP) extract and the biosynthesized silver and gold nanoparticles (Ag@UP and Au@UP, respectively) was assessed by determination of the minimum inhibitory concentration (MIC) and minimum bactericidal concentration (MBC). Both the MIC and MBC were determined against *Staphylococcus aureus* ATCC 6538 (gram-positive), *Pseudomonas aeruginosa* ATCC 10145 (gram-negative), and *Escherichia coli* ATCC 11303 (gram-negative), in accordance with EUCAST/CLSI recommendations [9,48]. In all assays, silver nitrate, kanamycin (an aminoglycoside antibiotic), and ampicillin (a β-lactamic antibiotic) were used as positive controls. For the determination of the MIC, 50 µL of bacterial suspensions in a Mueller–Hinton Broth (MHB; 1 × 10^6^ CFUs/mL) were mixed with 50 µL of serial diluted testing samples (between 140 and 1700 µg/mL for the UP extract; 0.54 and 11.81 µg/mL for Ag@UP and Au@UP; 0.85 and 10.19 µg/mL for silver nitrate; 5 and 60 µg/mL for kanamycin and ampicillin) in 96-well plates and incubated overnight at 37 °C. Growth inhibition was determined by measuring the optical density at 600 nm, and the MIC was defined as the lowest concentration where no visible growth was observed. For the MCB assays, 10 µL of each sample was diluted 1:100 (*v/v*) in saline solution and plated into a MHB solid medium, followed by incubation at 37 °C for the enumeration of CFUs.

### 3.7. Inhibition of Biofilm Formation

The ability of the silver (Ag@UP) and gold (Au@UP) nanoparticles to inhibit biofilm production was assessed as previously described [48] against *S. aureus* ATCC 23235 and *P. aeruginosa* PAO1, which have a mucoid phenotype and are capable of producing biofilms [63]. Briefly, 50 μL of bacterial cell cultures grown overnight in MHB (1 × 10^6^ CFUs/mL) were mixed with 50 μL of serial diluted testing samples (using the same range of concentrations described in the section above) in 96-well plates and incubated overnight at 37 °C. Bacterial cell cultures without nanoparticles were used as positive controls for biofilm formation. After incubation, the medium was carefully discarded, and wells were gently washed with sterile phosphate buffered saline (PBS) to remove non-adhered cells (planktonic). The adhered cells were fixed with 200 µL of methanol anhydrous 99.8% for 5 min. Subsequently, the methanol was removed, and cells were stained with 200 µL of 0.2% crystal violet for 5 min, followed by three washing steps with PBS. The crystals were solubilized with acetic acid (33% in PBS), and the absorbance was read at 570 nm. The MIC for biofilm formation was determined as the concentration at which the absorbance reached negligible levels (i.e., 100% of inhibition) compared to the control (bacterial cell cultures without nanoparticles).

### 3.8. Antifungal Assays

Antifungal assays were performed through the determination of the minimum fungicidal concentration (MFC) against two species of *Candida albicans* (*C. albicans 124a* and *C. albicans SC5314*) and two species of *Candida auris* (*Candida auris* 17-270 and *Candida auris* 17-274), following EUCAST/CLSI recommendations [9,64]. Fungal cell cultures were grown in a RPMI 1640 medium and diluted to a final density of 3 × 10^5^ CFUs/mL in the RPMI 1640 medium. For the MFC assays, 100 μL of fungal suspensions in the RPMI 1640 medium and 100 μL of the serial diluted samples, using the same range of concentrations described in the antibacterial assays, were mixed in 96-well plates and incubated overnight at 30 °C. The MFC was determined as the lowest concentration with no visible growth by measuring the optical density at 530 nm.

## 4. Conclusions

In this study, gold and silver nanoparticles were successfully produced using the invasive macroalgae *Undaria pinnatifida* aqueous extract as the reducing and stabilizing agents. These nanoparticles are spherical with an average size of 6.8 ± 1.0 nm and 14.1 ± 2.8 nm for Au@UP and Ag@UP, respectively. The FTIR and HPLC results indicate that carbohydrates and proteins of the UP extract may participate in the synthesis and capping of the nanoparticles.

Taken together, these results support Au@UP and Ag@UP as novel nanomaterials exerting very strong antifungal effects, even at very low concentrations, with Ag@UP also exhibiting potent antibacterial activity. Furthermore, in addition to an environment-friendly synthetic process, this strategy proposes to exploit the abundance of a rapidly growing invasive macroalga species, *Undaria pinnatifida,* with positive prospects for bio-sustainability and biodiversity. This study points towards a biosustainable production of novel nanomaterials combining both antibacterial and antifungal potential.

## Figures and Tables

**Table 1:** MIC and MBC concentrations (µg/mL) determined for silver (Ag@UP) and gold (Au@UP) nanoparticles biosynthesized using the *Undaria pinnatifida* extract (UP extract) against *E. coli* ATCC 11303, *P. aeruginosa* ATCC 10145, and *S. aureus* ATCC 6538. Ampicillin, kanamycin, and silver nitrate were used as positive controls. Conditions where MIC or MBC was not reached are defined as not applicable (n.a.).

	*E. coli*		*P. aeruginosa*		*S. aureus*	
	MIC (µg/mL)	MBC (µg/mL)	MIC (µg/mL)	MBC (µg/mL)	MIC (µg/mL)	MBC (µg/mL)
**UP extract**	n.a.	n.a.	n.a.	n.a.	n.a.	n.a.
**Ag@UP**	1.96	3.94	1.96	3.94	1.96	3.94
**Au@UP**	n.a.	n.a.	n.a.	n.a.	n.a.	n.a.
**Ampicillin**	40	60	60	60	60	60
**Kanamycin**	60	60	60	60	60	60
**Silver nitrate**	10.19	10.19	10.19	10.19	6.79	10.19

**Table 2:** MIC concentrations (µg/mL) determined for silver (Ag@UP) and gold (Au@UP) nanoparticles biosynthesized using the *Undaria pinnatifida* extract (UP extract) against the biofilm-producing bacteria *P. aeruginosa* PAO1, and *S. aureus* ATCC 25923. Ampicillin, kanamycin, and silver nitrate were used as positive controls. Conditions where MIC was not reached are defined as not applicable (n.a.).

	*P. aeruginosa*	*S. aureus*
	MIC (µg/mL)	MIC (µg/mL)
**UP extract**	n.a.	n.a.
**Ag@UP**	7.88	7.88
**Au@UP**	11.81	11.81
**Ampicillin**	20	20
**Kanamycin**	20	20
**Silver nitrate**	4.31	2.16

**Table 3:** MFC concentrations (μg/mL) determined for UP extract, Au@UP, Ag@UP, and positive controls against *C. albicans* and *C. auris*. Conditions where MFC was not reached are defined as not applicable (n.a.).

	*Candida albicans* *124a*	*Candida albicans SC5314*	*Candida auris 117*	*Candida auris 120*
	MFC (µg/mL)	MFC (µg/mL)	MFC (µg/mL)	MFC (µg/mL)
**UP extract**	n.a.	n.a.	n.a.	n.a.
**Ag@UP**	0.54	1.97	0.54	0.54
**Au@UP**	0.54	n.a.	0.54	0.54
**Silver nitrate**	n.a.	6.79	4.31	n.a.

## Data Availability

Data will be made available upon reasonable request.

## Supplementary Materials

The following are available online at https://www.mdpi.com/article/10.3390/md21070397/s1, Figure S1: Representative images of *Candida sp* in the presence (A, B) and absence (C, D) of the nanoparticles. Size bars correspond to 500 µm; Figure S2: Hyphae growth assay in different Candida species (*C. albicans 124a (A)*, *C. albicans SC5314 (B)*, *C. auris 17-274 (C)*, *C. auris 17-270 (D)*) expressed against the growth control after 24 h of incubation with Ag@UP, Au@UP, and UP extract. Results are shown as a percentage of growth in relation to the life control (only medium); Figure S3: Image of *Undaria pinnatifida* (Harvey) Suringar 1873 (published in [20]).
