# Supplementary material for: Valorisation of the Invasive Macroalgae Undaria pinnatifida (Harvey) Suringar for the Green Synthesis of Gold and Silver Nanoparticles with Antimicrobial and Antioxidant Potential
Source: Mar Drugs. 2023 Jul 9;21(7):397. doi: 10.3390/md21070397 (PMC10381743; doi:10.3390/md21070397)
Supplement: Supplementary file 1 [file marinedrugs-21-00397-s001.zip › marinedrugs-2454656-supplementary.pdf]

Supplementary Materials

For the verification of hyphae production by *C. albicans 124a*, *C. albicans SC5314*, *Candida auris* 17-270 and *Candida auris* 17-274, with and without the incubation of the extracts and nanoparticles (0.54 µg/mL was identified as the MFC of Ag@UP and Au@UP against *C. albicans* 124a and both *C. auris* strains; for reference strain *C. albicans* SC5314, MFC were 1.97 µg/mL for Ag@UP and >11.81 µg/mL for Au@UP), the *Candida* were grown in Dulbecco's Modified Eagle Medium (DMEM) supplemented with 10% fetal bovine serum (FBS). They were then incubated with the compounds and after 24h the cells were fixed in a 70% (v/v) ethanol solution and visualized at 40X objective (**Figure S1** and **Figure S2**). All results are expressed in percentage, in relation to the hyphae growth observed in life control samples.

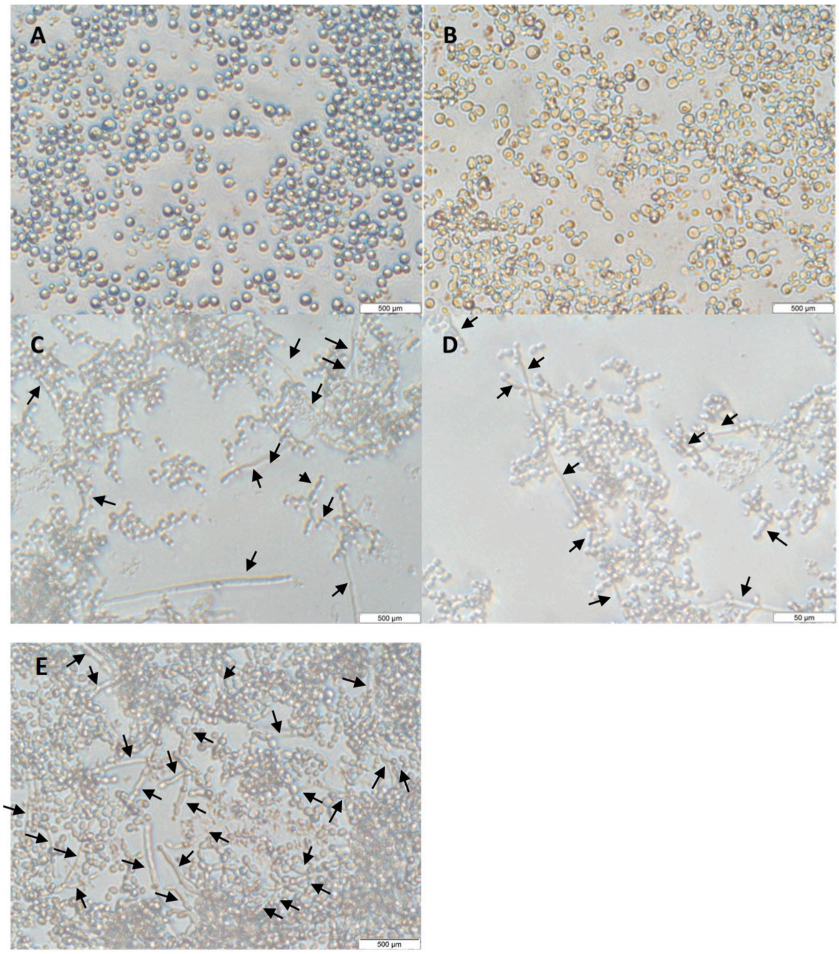

Figure S1: Representative images of *Candida* sp in the presence (A, B) and absence (C, D, control E) of the nanoparticles. Forming hyphae are identified by an arrow head. Size bars correspond to 500 µm.

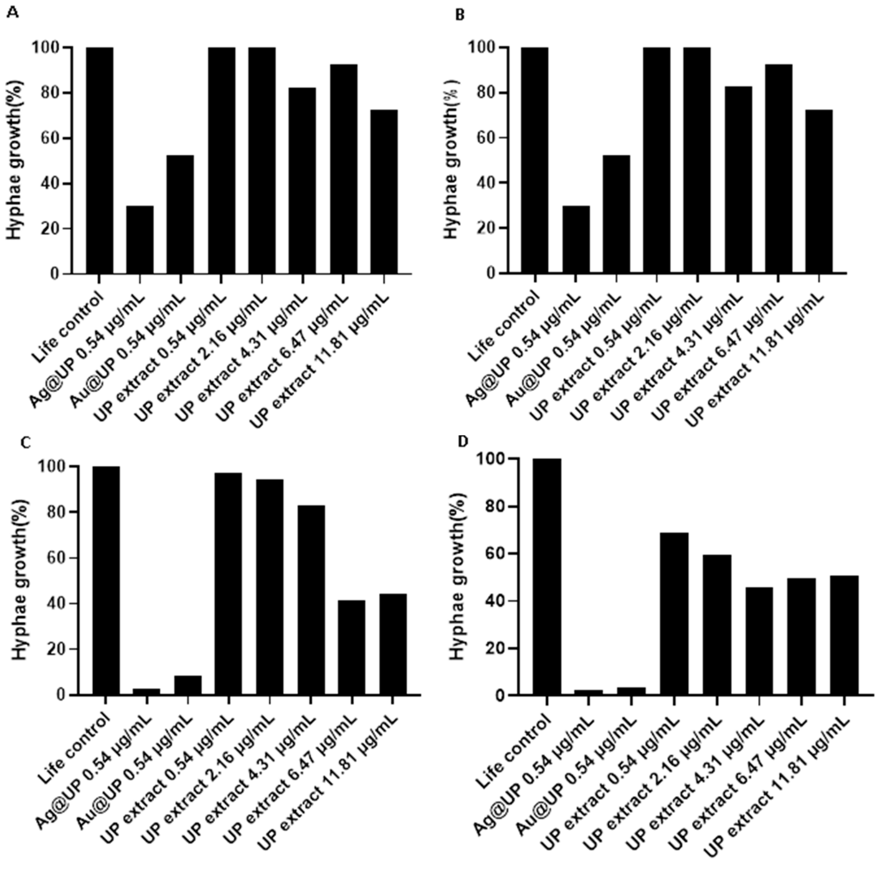

Figure S2: Hyphae growth assay in different *Candida* species *(C. albicans 124a (A)*, *C. albicans SC5314 (B), C. auris 17-274* (C), *C. auris 17-270 (D))* expressed against the growth control after 24 h of incubation with Ag@UP, Au@UP and UP extract. Results are shown as a percentage of growth in relation to the life control (only medium).

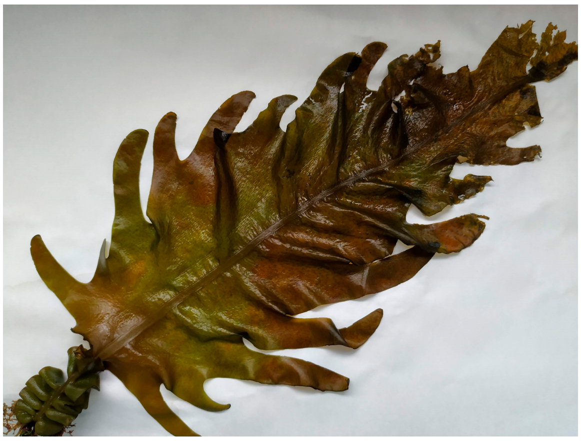

Figure S3- Imagen of *Undaria pinnatifida* (Harvey) Suringar 1873 (published in [20]).
